# Improving mental health service users’ with medical co-morbidity transition between tertiary medical hospital and primary care services: a qualitative study

**DOI:** 10.1186/s12913-016-1567-3

**Published:** 2016-07-26

**Authors:** Kate Cranwell, Meg Polacsek, Terence V. McCann

**Affiliations:** 1 Community Services, Western Health, Melbourne, VIC Australia; 2 Centre for Chronic Disease, College of Health and Biomedicine, Discipline of Nursing, Victoria University, PO Box 14428, Melbourne, VIC 8001 Australia

**Keywords:** Service users’ and caregivers’ experience, Experience-based co-design, Mental Health Hospital Admission Reduction Program, Medical co-morbidity, Transition

## Abstract

**Background:**

Mental health service users have high rates of medical co-morbidity but frequently experience problems accessing and transitioning between tertiary medical and primary care services. The aim of this study was to identify ways to improve service users’ with medical co-morbidity care and experience during their transition between tertiary medical hospitals and primary care services.

**Method:**

Experience-based co-design (EBCD) qualitative study incorporating a focus group discussion. The study took place in a large tertiary medical service, incorporating three medical hospitals, and primary care services, in Melbourne, Australia. A purposive sample of service users and their caregivers and tertiary medical and primary care clinicians participated in the focus group discussion, in August 2014. A semi-structured interview guide was used to inform data collection. A thematic analysis of the data was undertaken.

**Results:**

Thirteen participants took part in the focus group interview, comprising 5 service users, 2 caregivers and 6 clinicians. Five themes were abstracted from the data, illustrating participants’ perspectives about factors that facilitated (clinicians’ expertise, engagement and accessibility enhancing transition) and presented as barriers (improving access pathways; enhancing communication and continuity of care; improving clinicians’ attitudes; and increasing caregiver participation) to service users’ progress through tertiary medical and primary care services. A sixth theme, enhancing service users’ transition, incorporated three strategies to enhance their transition through tertiary medical and primary care services.

**Conclusion:**

EBCD is a useful approach to collaboratively develop strategies to improve service users’ with medical co-morbidity and their caregivers’ transition between tertiary medical and primary care services. A whole-of-service approach, incorporating policy development and implementation, change of practice philosophy, professional development education and support for clinicians, and acceptance of the need for caregiver participation, is required to improve service users’ transition.

## Background

Mental health service users (service users) have high rates of medical co-morbidity [1], and medical co-morbidity and mortality occur more often in this population than the general population [2]. In particular, medical co-morbidity can lead to a more acute form of mental illness, reduced quality of life and premature mortality [3–5]. A United States study found that, compared to the general population, people with severe mental disorders had a significantly higher prevalence of medical co-morbidity in 14 out of 17 medical conditions assessed [6]. The main causes of service users’ reduced life expectancy are untimely cerebrovascular and coronary heart diseases [7] with upwards of 60 % of this mortality being due to routine preventable and treatable medical illnesses [8]. For instance, type-2 diabetes mellitus and diabetogenic complications are more common in people with schizophrenia in comparison to the general population [9], and medical co-morbidity causes 60 % of non-suicide-related premature mortality in this cohort [10]. Even though service users have a high prevalence of medical co-morbidity, they regularly experience difficulties accessing and obtaining appropriate treatment for these conditions from public health services [10]. Inability to access timely treatment for, and a higher prevalence of, medical co-morbidity is likely to lead to greater mortality and premature death in this stigmatised and disadvantaged group of individuals [11].

Despite the fact that the association between mental illness and medical co-morbidity is well-known, the latter continues to be diagnosed and treated inadequately [3]. Four main influences affect the onset and progression of medical co-morbidity. (i) Consumer-related influences: Adverse cognitive and communication effects of the consumer’s mental disorder, unhealthy dietary consumption and lifestyle, and a potential genetic susceptibility to medical illness [3]. (ii) Help-seeking and help-receiving experience-related influences: Service users’ belated or absence of attempts to seek help, which may be due to poor health literacy, community and clinicians’ stigmatisation of people with mental illness [12], and service users’ dissatisfaction with the treatment received [13]. To illustrate the influence of stigma in health professionals, senior physicians in Norway ranked schizophrenia and depression 34th and 35th respectively out of 38 conditions they least like to treat [14]. (iii) Access and treatment-related influences: Inadequate access to primary and tertiary medical care; sub-optimal medical care, as well as under-diagnosis and insufficient treatment of medical illness [3, 15, 16]. This situation may also be attributable to general practitioners’ (GPs) [11] and hospital clinicians’ [17] hesitancy, lack of understanding and expertise in treating people with serious mental illness. For example, a review of literature of general medical nurses’ attitudes toward, and care of, service users with medical co-morbidity concluded that there was considerable room for improvement in nurses’ empathetic attitudes and competent care [18]. Another treatment-related influence is ‘diagnostic overshadowing’ or attributing medical symptoms wrongly to existing mental illness, which can lead to under or delayed diagnosis and incorrect or delayed medical treatment [19]. (iv) Harmful side effects of psychotropic medications: Metabolic syndrome and metabolic abnormalities, such as weight gain, hyperglycaemia and dyslipidaemia, significantly increase in prevalence in service users treated with psychotropic medication [4, 20].

A potential way of bridging the gap between service users with medical co-morbidity (and their primary caregivers [caregivers]) and their access to, and receipt of appropriate care in tertiary medical hospitals, is to adopt a co-design approach. Although still an emerging approach, co-design entails a partnership approach between service users, caregivers and clinicians [21]. One co-design approach is experience-based co-design (EBCD), a service user-centred (experience-based) participatory action research approach, which can lead to collaborative change (co-design) in service provision between service users and clinicians [21]. EBCD is a staged approach in which service users, caregivers and clinicians work together to identify, implement and evaluate improvements to service delivery [22]. Integral to this process is clinicians striving to understand service users’ and caregivers’ lived experience of healthcare service delivery [23]. By reconceptualising their role EBCD provides a participatory framework to enhance quality improvement processes [21]. When used successfully, the approach enables healthcare providers to understand what works well, identify areas where service users’ and caregivers’ care and experiences can be improved, identify areas where professional development of the workforce is required, and transform organisational systems to enhance service users’ and caregivers’ care and experiences. EBCD was established in the United Kingdom by the King’s Fund for the National Health Service and trialled in a head and neck cancer service in England in 2005. While the approach has been adopted in healthcare settings in other countries, its use is comparatively new in Australia [21].

Because of increasing attention being placed on meeting service users’ needs with medical co-morbidity [3, 6], an EBCD approach may help shed light on the strategies needed to improve their transition between tertiary medical hospitals and primary care services. Therefore, the purpose of this study was to identify ways to improve service users’ with medical co-morbidity care and experience during their transition between tertiary medical hospitals and primary care services. The objectives were to highlight transition enablers and barriers, and identify strategies to improve service users’ and caregivers’ transition. The study was nested within a larger EBCD study of the experience of service users with medical co-morbidity (and their caregivers) as they transitioned between tertiary medical hospitals and primary care services. The larger study was conducted in five-stages:

1. Video-recorded service user and caregiver interviews. These were held to capture service users’ (*n* = 12) and caregivers’ (*n* = 4) experience of their transition. The findings provided four main themes that reflected their experience of the transition: 1. accessing tertiary medical hospital services was difficult and time-consuming, 2. contrasting experiences of clinician engagement and support, 3. lack of continuity of care between tertiary medical and primary care services, and 4. Mental Health Hospital Admission Reduction Program (MH HARP) (an augmented care program to reduce the number of emergency department re-presentations by service users with medical co-morbidity) clinicians facilitated service users’ transition [24].
2. Clinician focus group discussion and individual interviews. Two focus group discussions were held with mental health clinicians (*n* = 17). Individual interviews were conducted with GPs (*n* = 4), to ascertain their perspectives about the transition.
3. Production of a professionally edited 20-min film containing video clips of commentary by service users and caregivers, key quotes from service users, caregivers and clinicians, and voiceover that best illustrated the themes arising from stages 1 and 2 of the project.
4. Combined focus group discussion (the subject of the present paper) comprising service users and their caregivers and tertiary medical and primary care clinicians.
5. Evaluation survey of service user, caregiver and clinician participants’ experiences and views about the effectiveness of the EBCD process.

## Method

### Design

A focus group discussion approach to data collection was used because it is an ideal method for drawing on the interaction between participants with different perspectives, experiences, attitudes, beliefs and feelings, about a phenomenon [25], in this instance, service users with medical co-morbidity, their caregivers and tertiary medical hospital and primary care clinicians. Although it is recommended that a focus group should contain six to eight participants, the size is dependent on the topic, how much participants know about the topic and their availability, and the facilitator’s expertise in guiding and facilitating the discussion [26]. Data collection for the complete project was undertaken between March and August 2014, and for the combined focus group discussion, in August 2014.

### Sample and recruitment

A purposive sample of service users and their caregivers, who participated in Stage 1 of the study, were recruited through the tertiary medical service’s MH HARP, an augmented care program, in Melbourne, Australia. MH HARP is an initiative of the Council of Australian Governments (COAG) [27]. The program was introduced as a four-year pilot scheme, in 2013; the scheme operating in this tertiary medical service was being undertaken in the state of Victoria. MH HARP was introduced for service users with medical co-morbidity who had at least two presentations at the tertiary medical service’s emergency departments within the previous 12 months. The program offers these service users intensive care coordination and support for up to six months, including enhancing their capacity to self-manage their co-morbidities and connecting them with suitable community services.

MH HARP clinicians (mainly mental health nurses) contacted service users and their caregivers to ascertain their provisional interest in participating in the present study. If interested, a researcher contacted them, outlined the study, answered their questions and obtained consent. Purposive sampling was adopted to assist data collection [28]. Inclusion criteria were: (i) service users with acute and severe medical co-morbidity and their caregivers; (ii) two or more emergency department presentations within the preceding 12 months, (iii) previous participants in MH HARP; (iv) aged over 18 years; and (v) able to communicate in conversational English. The exclusion criterion was: a consumer currently undergoing an acute episode of medical or mental illness.

A purposive sample of experienced clinicians who participated in Stage 2 of the study was recruited from MH HARP, two mental health services, and GP practices. All clinician participants were involved in direct clinical practice with service users.

### Data collection

TMcC, an expert in qualitative research, moderated the focus group, and KC and MP took detailed hand-written notes of the key points discussed. The meeting took place, in private, in a large room in the tertiary medical service’s education and research complex. The interview format was semi-structured, lasted 3 h, and included a short food and refreshment break. A semi-structured focus group interview guide was developed from key findings of stages 1–3 of the project. Three broad questions framed the discussion: (i) What works well in the transition process for service users? (ii) What difficulties are encountered in the transition process? (iii) What specific strategies should be adopted to improve this process? Answers to questions were probed and scrutinised further. At the conclusion of each segment of the interview, the researcher, assisted by the use of whiteboards, summarised participants’ responses to ensure their experiences and beliefs were captured and understood correctly, a validation process that strengthened the credibility of the study [29].

There were 4 parts to the focus group discussion:

1. Introduction and overview. Participants were welcomed. The moderator (TMcC), two co-researchers (KC and MP) and participants introduced themselves. A summary of the aims and stages of the project and an overview of the proceeding of the focus group meeting was provided. Participants were requested to maintain confidentiality about issues discussed within the focus group.
2. View the 20-min video (Stage 3 of the project referred to earlier).
3. Using the video as a basis for discussion, identify (a) what works well in the transition process, (b) broad areas for improvement in the process, and (c) specific areas for improvement in this process. Whiteboards were used to facilitate this activity and provide participants with a visual reference of discussion points and agreement.
4. Negotiate co-design initiatives.

### Data analysis

Cognisant of the overarching framework in which the questions were asked (facilitators, barriers and transitions), we adopted Braun and Clarke’s ([30], p.79) six-step approach to thematic analysis. (1) *Familiarisation with the data.* Data were transcribed and read and re-read to gain a broad appreciation of participants’ perspectives about service users’ and caregivers’ transition. (2) *Generating initial codes.* Transcripts were scrutinised and initial were codes inserted manually. (3) *Searching for themes*. Codes were clustered into provisional themes. (4) *Reviewing themes.* Themes were appraised to establish if they worked in relation to the coded extracts, and a thematic ‘map’ of the analysis was developed. (5) *Defining and naming themes.* On-going analysis, naming, refinement and ordering of themes took place. Saturation of themes with ‘thick’ description of the data occurred when no new data was identified to contribute to each theme [31]. Simultaneously, data reduction took place with provisional themes inadequately supported by data being omitted. (6) *Producing the report.* Selection of illustrative exemplars for each theme and producing a scholarly report occurred.

Preliminary thematic analysis was carried out by KC and MP. This was followed by an independent review of the process by TMcC, an activity that improved the rigour of the study [28]. Differences in coding and theme identification were overcome through discussion. A semantic level of analysis was conducted, proceeding from description and summary, in the results section, to interpretation and discussion, in the discussion section [30].

### Ethics

Ethical approval to undertake the study was given by Melbourne Health Human Research Ethics Committee (MH2013.255) and the tertiary medical service’s Office for Research. Service users (and caregivers) were informed that refusal to participate, or to take part but subsequently withdraw from the study, would have no adverse effects on their current or future care or, in the case of clinicians, their employment. All participants provided written consent to participate.

### Rigour

We adopted four criteria to ensure rigour in the study: dependability, confirmability, credibility and transferability [31]. Dependability and confirmability were maintained by devising an audit trail linking raw data and codes with themes. Furthermore, preliminary thematic analysis was undertaken by KC and MP, followed by an independent review of the process by TMcC [28]. Credibility was enhanced by using a semi-structured interview guide to ensure continuity of focus was maintained with the first three stages of the study and that a wide range of participants’ experiences were represented in the combined focus group data [32]. Credibility was also strengthened by participant verification, which involved summarising or paraphrasing participants’ comments to ensure their comments were understood correctly [29]. Transferability was maintained by presenting sufficient exemplars in the results section to support the themes. By evaluating the process and results, readers may also assess their transferability to other similar contexts [31].

## Results

Thirteen participants took part in the focus group discussion, comprising 5 service users (4 females, 1 male) and 2 caregivers (1 male, 1 female) who participated in the filmed interviews (Stage 1), 5 experienced mental health clinicians (2 females, 3 males) who took part in the clinician focus groups (Stage 2) and 1 GP (male) who participated in the individual interviews (Stage 2).

Five themes were abstracted from the data highlighting service users’, caregivers’ and clinicians’ perspectives about factors that enabled (clinicians’ expertise, engagement and accessibility enhancing transition) and hindered (improving access pathways; enhancing communication and continuity of care; improving clinicians’ attitudes; and increasing caregiver participation) service users’ progress through tertiary medical and primary care services. A sixth theme, adopting transition enhancing strategies, encompassed strategies to improve their transition between tertiary medical and primary care services.

### Facilitators

#### Clinicians’ expertise, engagement and accessibility enhancing transition

Service users and caregivers commented that some MH HARP clinicians displayed high standards of mental health and interpersonal skills, and perceived them as ‘experts’ in their field of practice. These participants highlighted the centrality of clinicians being able to engage and develop professional relationships with clinical staff.

*I found just talking on the phone for a few minutes really helped* (Participant 1; female service user).

Similarly, they commented favourably about the importance of having helpful, supportive and accessible general practitioners (GPs) to facilitate service users’ transition.

*Everything was put in place. My GP is helping. I know now that I can access help* (Participant 6; male service user).

### Barriers

Service users, caregivers and clinicians identified several system and personnel-related barriers that needed to be overcome in order to improve service users’ transition, and these are reflected in the following four themes: (i) improving access pathways, (ii) enhancing communication and continuity of care, (iii) improving clinicians’ attitudes, and (iv) increasing caregiver participation.

#### Improving access pathways

Service users and caregivers commented that it was difficult for them to access tertiary medical hospital services because the multiple access pathways were confusing and complicated to navigate and most were associated with long waiting times. Clinicians attributed service users’ and caregivers’ access difficulties to geographical boundaries, based on the service user’s place of residence, which enabled or denied access to services, and service users and caregivers having to navigate through different layers of bureaucracy in tertiary medical services. In attempting to navigate these multiple pathways, service users were frustrated by the duplication of assessment of their medical conditions at every point in the continuum of care. Moreover, on some occasions their referrals were not followed up and they ‘fell through the gaps’ in the services, with the consequence that they failed to receive their relevant appointments in the services.

*You are re-assessed everywhere you go* (Participant 4; female service user).

*You re-tell your story over and over again* (Participant 5; female service user).

#### Enhancing communication and continuity of care

Service users and caregivers expressed frustration about their service expectations not being fulfilled and about poor service coordination. This was especially the case with emergency department staff, including mental health clinicians in these departments. From these participants’ perspectives, clinicians seemed to be working under constant pressure and, as a result, were primarily crisis-focused or focused on dealing with the immediate problem. However, once the initial problem was dealt with there was also need for clinicians to place greater emphasis on rehabilitation and continuity of care.

*I stopped asking as there was a feeling that services are under pressure* (Participant 3; female caregiver).

*Services are few and far between. It is a big letdown* (Participant 7; male caregiver).

Participants also commented about the way clinicians communicated with them. They remarked about the general lack of information and poor communication from clinicians as well as their use of clinical jargon, which heightened service users’ and caregivers’ confusion and uncertainty. They claimed that usually they were not informed about what to expect from services and about their treatment and care options. They also commented that they needed better communication to assist them to access and navigate the services, including connecting with case managers. Similarly, there was a need to improve communication related to service users’ continuity of care. This entailed increasing information and communication between tertiary and primary care services and following up to ensure that service users’ transition to a new service had taken place satisfactorily. There was also a need to provide long-term case management. The GP participant also emphasised the benefits of maintaining contact with service users before, during and after and the episode of contact with the tertiary medical service. Regarding MH HARP clinicians, while their overall impression of services provided by these clinicians was favourable, service users and caregivers commented that they had insufficient contact with these clinicians and would benefit more so if the program was extended beyond the current 6-month timeframe.

#### Improving clinicians’ attitudes

Service user and caregiver participants commented about the negative attitudes, sarcastic comments, and discriminatory behaviour of tertiary medical and mental health service clinicians towards them. They perceived that clinicians responded to them in ways that suggested service users were making false claims about their medical condition and engaging in attention seeking behaviour. They pointed out the need for clinicians to improve these attitudes and behaviours. Service users and caregivers also claimed that many mental health clinicians appeared to be ‘burnt out’ or exhausted from overwork.

*They treated me like I was making things up, and I just wanted their attention* (Participant 5; female service user).

Generally, service users and caregivers felt clinicians needed to be more empathetic, especially in emergency departments and community mental health services. They commented that professional development training should be provided to clinicians about how to communicate with and respond to people with mental health problems because they seemed to be unable to deal effectively with service users with these problems is settings such as emergency departments.

*There is not enough compassion in emergency departments and mental health services* (Participant 4; female service user).

*The way you are communicated with is not good* (Participant 2; female service user).

#### Increasing caregiver participation and support

Caregivers commented that clinicians undervalued their contribution as carers; that they often felt forgotten, overlooked and frustrated. The frustration led caregivers to give up trying to seek assistance from clinicians. In contrast, they claimed they should have a more prominent role in decision-making, especially in goal setting and discharge planning about their family member service user. They also stated they needed to be provided with more information about the various forms of support available to caregivers in their situation.

*A pivotal thing for me was how often I was left out of discussions and decision-making.* (Participant 3; female caregiver).

*You give up.... I could have done so much more myself* (Participant 3; female caregiver).

### Transition

#### Adopting transition enhancing strategies

Having abstracted four themes highlighting broad areas for improvement in service users’ and caregivers’ transition between tertiary medical and primary care services (improving access pathways, enhancing communication and continuity of care, improving clinicians’ attitudes, increasing caregiver participation), the theme adopting transition enhancing strategies encompassed three pragmatic approaches to facilitating this transition. In identifying these strategies, consideration was given to the scope and timeframe of the present EBCD project and, moreover, that the co-design initiatives were achievable and of immediate practical and tangible benefit to current and future service users, caregivers and clinicians.

##### Produce a service user information brochure about MH HARP

In order to improve service users’ (and caregivers’) understanding of, and facilitate their navigation through, tertiary medical and primary care services, focus group participants agreed that a brochure should be designed and introduced to facilitate this process. The outcome of this proposal was that a new brochure was developed that explained, in plain language, the rationale for, operation of, and how the MH HARP service could assist service users. A direct access telephone number was included in the brochure to enhance service users’ and caregivers’ access to MH HARP clinicians to discuss issues. The brochure is now provided to all new service users referred to the service.

##### Improve continuity of care by introducing post-discharge follow-up

Lack of continuity of care was identified as a major barrier to service users’ transition between tertiary medical and primary care services. Focus group participants agreed that it was important to design and implement a consistent post-discharge follow-up process in order to improve continuity of care. The outcome of this proposal was that MH HARP clinicians now contact service users by telephone within four weeks of discharge from the tertiary medical service, to ensure that arrangements made at the time of discharge have supported their continuity of care. In addition, service users and caregivers are now asked to provide feedback about this aspect of service provision to MH HARP clinicians, and their comments are included as a standard item on the agenda of regularly scheduled team meetings of MH HARP clinicians.

##### Increasing service awareness of MH HARP

As MH HARP was a relatively new initiative, focus group participants agreed that it was necessary to increase awareness and understanding of this initiative throughout tertiary medical and primary care services. In so doing, participants felt this would benefit service users’ transition directly and indirectly. The outcome of this proposal was that meetings were held with key tertiary medical and primary care service stakeholders, to establish opportunities to improve communication and streamline referral between MH HARP and regional mental health service providers.

## Discussion

The aim of this qualitative study was to identify ways to enhance mental health service users’ with medical co-morbidity care and experience during their transition between tertiary medical hospitals and primary care services. In particular, we sought to establish what worked well in this process, ascertain transition barriers, and identify strategies to improve service users’ transition. Service users and caregivers experienced several transition facilitators such as some MH HARP clinicians’ high standards of mental health and interpersonal skills. At the same time, they encountered several transition barriers and identified ways to overcome these barriers. They highlighted the need to improve access pathways to services because of difficulties they encountered accessing services. They emphasised the need for better communication and continuity of care, especially by emergency department staff and mental health staff deployed in these departments.

Service user and caregiver participants in the current study emphasised the need to improve clinicians’ communication, attitudes and behaviours as they felt they were given insufficient information, treated in discriminatory ways and their medical concerns were not taken seriously. Stigmatisation by clinicians and dissatisfaction with treatment can have detrimental implications for service user help-seeking and engagement with services [12, 13, 33]. The implication of clinicians’ negative attitudes toward people with mental disorder is poorer quality of care and, indirectly, undiagnosed or poorly treated medical [13, 19]. Poor quality of care in areas such as under-diagnosis, insufficient treatment of serious illnesses [3, 15], ‘diagnostic overshadowing’ (misattributing medical symptomatology to co-existing mental illness) [13, 19] and medical clinicians’ [10, 17] unwillingness to care for service users with mental disorder can culminate in under or delayed diagnosis and inappropriate or delayed treatment of medical co-morbidity [13, 19]. Ways to decrease stigma and, as a consequence, increase help-seeking include challenging stigma in health services [34] and, in the present study, increasing the scope of practice of MH HARP clinicians to enable them to bridge the gap between emergency department clinicians and service users and caregivers and in providing continuing professional development education and training to this group of clinicians.

In the present study, caregivers felt that their contribution, as carers, was undervalued, and claimed that they should be given a more prominent role in decision-making and receive more support from clinicians in their caregiving role. The issue of caregivers perceiving their role is undervalued and being excluded from clinical decision-making about the person with schizophrenia is commensurate with the findings of two other studies. A Swedish study of caregivers of people with mental disorder, by Ewertzon et al. [35], reported that carers perceived clinicians as uncooperative and unappreciative of their contribution, and an Australian study, by McCann et al. [36], of caregivers of young people with first-episode psychosis, found that caregivers perceived they were taken-for-granted and excluded from clinical deliberations by clinicians. Being undervalued and taken-for-granted may be attributable to clinicians’ stigmatising and superior attitudes, where caregivers’ considerable, but lay-informed, understanding is overlooked in favour of professional-based knowledge [37, 38].

Poor continuity of care was highlighted as problematic in the current study. The implication of poor continuity of care, in service users with medical co-morbidity, is it can lead to higher use of emergency departments and this, in turn, result in greater hospitalisation rates [39]. It is noteworthy that a primary reason for establishing MH HARP in Australia was to reduce the number of presentations to emergency departments by service users with medical co-morbidity [27]. Another consideration is that high rates of emergency department presentations and/or medical hospitalisation — illustrating a high prevalence of medical co-morbidity in this population — highlight the need to better integrate and embed medical care for service users in psychiatric inpatient and community settings [16]. Two of the strategies implemented as co-design initiatives in the present study — the brochure and providing greater continuity of care and post-discharge follow-up — may help reduce the rates of emergency department representations and hospitalisations.

### Study limitations

Our study has four limitations. First, as a qualitative study the findings are limited to the participants and services in which the study was undertaken [40]. Even though generalisability is not a primary consideration while conducting qualitative research [41], the themes can be validated [42, 43] and are applicable to service users with medical co-morbidity and caregivers in other contexts transitioning between tertiary medical and primary care services. Second, MH HARP clinicians who facilitated service user and carer recruitment may have been selective in recruiting participants. Third, service user and caregiver participants who were not part of MH HARP may have a different experience of this transition. Finally, only one GP participated; other GPs may have had different perspectives about the issues discussed.

## Conclusion

Overall, the three main co-design strategies — producing a brochure for service users, providing greater continuity of care and post-discharge follow-up, and increasing awareness of MH HARP — are worthwhile initiatives, which may help overcome some transition barriers for service users and caregivers. However, research is needed to evaluate if these co-design strategies are effective in reducing emergency department presentation and hospitalisation rates. Furthermore, more needs to be done to address other transition barriers such as improving clinicians’ communication, attitudes and the ways they deal with service users, and in enhancing caregiver participation in decision-making and support. As such, these require a whole-of-service approach, incorporating policy development and implementation; change of practice philosophy; professional development education and support for clinicians; and acceptance of the need for caregiver participation, and acknowledgement of their contribution to the care and support of the person with mental disorder.

## Abbreviations

Caregivers, primary caregivers; EBCD, experience-based co-design; MH HARP, Mental Health Hospital Admission Reduction Program; Service users, Mental Health Service Users
